# Alcohol and breast cancer

**DOI:** 10.1007/s43440-022-00426-4

**Published:** 2022-10-30

**Authors:** Beata Starek-Świechowicz, Bogusława Budziszewska, Andrzej Starek

**Affiliations:** Department of Biochemical Toxicology, Chair of Toxicology, Medical College, Jagiellonian University, Medyczna 9, 30-688 Kraków, Poland

**Keywords:** Ethanol metabolism, Breast cancer, Breast cancer risk factors, Alcohol and oestrogens, Preventive factors

## Abstract

Breast cancer is one of the main causes of death in women worldwide. In women, breast cancer includes over half of all tumours caused by alcohol. This paper discusses both ethanol metabolism and the mechanisms of mammary tumourigenesis caused by alcohol. Numerous signalling pathways in neoplastic transformation following alcohol consumption in women have been presented. In addition, primary and secondary prevention, phytochemicals, synthetic chemicals, specific inhibitors of enzymes and selective receptor modulators have been described.

## Introduction

Alcohol is the most consumed stimulant worldwide. Alcohol consumption has been related to different types of neoplasms in humans. There is epidemiological evidence that drinking alcohol elevates the risk of tumours in many organs, e.g. the oral cavity, larynx, oesophagus, liver, and pancreas. An increased risk of colorectal cancer has been observed in numerous studies [1].

Alcohol consumption is a well-established risk factor for breast cancer in women. Breast cancer is a disease with an aetiology that includes dietary, lifestyle, and hormonal risk factors. Among these factors, alcohol consumption has been related to higher breast cancer risk [2].

Globally, it was found that in 2016, there were 3.0 million yearly alcohol-attributable deaths (95% uncertainty interval, UI 2.6–3.6) and 131.4 million disability-adjusted life years (DALYs) (UI 119.4–154.4). This corresponds to 5.3% of all deaths (UI 4.6–6.3) and 5.0% of all DALYs (UI 4.6–5.9). Alcohol consumption was a major risk factor for communicable, maternal, perinatal, and nutritional diseases, with a population-attributable fraction (PAF) of 3.3% (UI 1.9–5.6), noncommunicable diseases 4.3% (UI 3.6–5.1), and deaths 17.7% (14.3–23.0). Diseases caused by alcohol were more frequent among men than among women. The alcohol-attributable diseases were highest in Eastern Europe and the western, southern, and central sub-Saharan Africa regions as well as in countries with low human development indices (HDIs). A total of 52.4% of all alcohol-attributable deaths occurred in people younger than 60 years old [3, 4].

Over 2 million new cases of breast cancer are detected annually worldwide. Globally, the prevalence of breast cancer is 6.8 million cases. There is a consistent opinion that the intake of as little alcohol as less than 10–15 g per day leads to an elevated risk of breast cancer [5].

It was demonstrated that in Europe in 2016, there were almost 80,000 cancer deaths caused by alcohol consumption (women 22,778; 95% confidence interval, CI 18,985–26,622; men: 56,207; 95% CI 54,142–66,709). In the same year, almost 1.9 million cancer disability-adjusted life years lost were estimated (women 531,377; 95% CI 450,307–614,370; men 1,349,113; 95% CI 1,306,596–1,591,898) [4].

Globally, the number of female deaths from breast cancer increased approximately 1.7 times (from 344,900 to 600,700 women) between 1990 and 2017. The age-standardized mortality rate (ASMR) of women’s breast tumours decreased by 0.59% (95% CI: 0.52–0.66%) annually. This decrease was mainly caused by the reduction in alcohol consumption and tobacco-associated female breast cancer [6].

While the number of female breast cancer deaths caused by alcohol consumption rose from 44,200 in 1990 to 56,800 in 2017, and the ASMR values were reduced from 2.04/100,000 to 1.33/100,000 in that time. In 2017, alcohol consumption accounted for approximately 10% of total deaths from female breast cancer worldwide. This value was high in Western Europe, Latin America, and Australasia. In some regions, the ASMRs of female breast cancer associated with alcohol consumption were markedly reduced. The greatest reduction was observed in North America. In most developing countries, in the regions of South Asia, Central Asia, and the Caribbean, the ASMRs of breast cancer were significantly increased. In some European countries, including Denmark (5.60/100,000), Serbia (5.00/100,000), and Luxemburg (4.90/100,000), the ASMRs of breast cancer were high. The greatest values of this proportion were observed in Namibia, Sri Lanka, and Vietnam [6]. Alcohol consumption is one of the main known risk factors for cancer in the European Union [4].

In a meta-analysis of prospective cohort studies, 22 cohorts and 45,350 breast cancer cases were analysed. Women who were drinkers at the time with oestrogen receptor positivity (ER +) had an elevated risk compared to non-drinkers. A relationship between total alcohol and wine dose consumption and increased breast cancer risk was observed. When consumin**g** 10 g alcohol per day, the risk increased by 10.5% (relative risk (RR) = 1.10; 95% CI: 1.08–1.13) in total alcohol and 8.9% (RR = 1.08; 95% CI: 1.04–1.14) in wine. For postmenopausal women, the risk increased by 11.1% (RR = 1.11; 95% CI 1.09–1.13) with every 10 g of the total alcohol increase. It was found that the breast cancer alcohol-associated percentage is higher in Europe than in North America and Asia. These data indicate that the effect of alcohol consumption on the incidence of breast cancer is mainly seen in ER + breast cancer. Quantitative analysis showed that drinking led to a significant risk of breast cancer, especially for postmenopausal women [7].

Similar results have been obtained in other studies. In the USA, 1484 cases of breast cancer were diagnosed (1190 invasive and 294 in situ) among 38,454 women. High alcohol consumption was related to a slight increase in breast cancer risk. For ≥ 30 g/day of alcohol versus none, the RRs were 1.32 (95% CI 0.96–1.82) for total breast cancer and 1.43 (95% CI 1.02–2.02) for invasive breast cancer. An elevated risk was limited to ER + and progesterone receptor (PR)-positive cancers. The RRs for an increase of 10 g/day of alcohol were 1.11 (95% CI 1.03–1.20) for ER + /PR + tumours (804 cases), 1.00 (95% CI 0.81–1.24) for ER + /PR − tumours (125 cases), and 0.99 (95% CI 0.82–1.20) for ER − /PR −  tumours (167 cases) [8].

In another study, comprising 184,418 postmenopausal women, 5,461 breast cancer cases were registered. Total breast cancer was significantly connected with alcohol intake, even in the case of a moderate amount of alcohol (> 10 g/day). In the case where the amount of alcohol was > 35 g, the RRs were 1.35 (95% CI 1.17–1.56) for total breast cancer, 1.46 (95% CI 1.22–1.75) for ductal tumours, and 1.52 (95% CI 0.95–2.44) for lobular tumours. The RRs for ER + /PR + , ER + /PR − , and ER − /PR −  tumours were 1.46 (95% CI 1.12–1.91) for > 35 g/day, 1.13 (95% CI 0.73–1.77) for 20 g/day, and 1.21 (95% CI 0.79–1.84) for above 20 g/day, respectively [9].

In women consuming 35–44 g per day, the alcohol RR of breast cancer was 1.32 (95% CI 1.19–1.45), whereas for women consuming ≥ 45 g per day, the alcohol RR of breast cancer was 1.46 (95% CI 1.33–1.61). The RR of breast tumours increased by 7.1% (95% CI 5.5–8.7%) for each additional 10 g of alcohol intake per day. In women with breast cancer and controls who did not consume alcohol, smoking did not raise the risk of breast cancer [10].

## Ethanol metabolism and its consequences

**Fig. 1:**
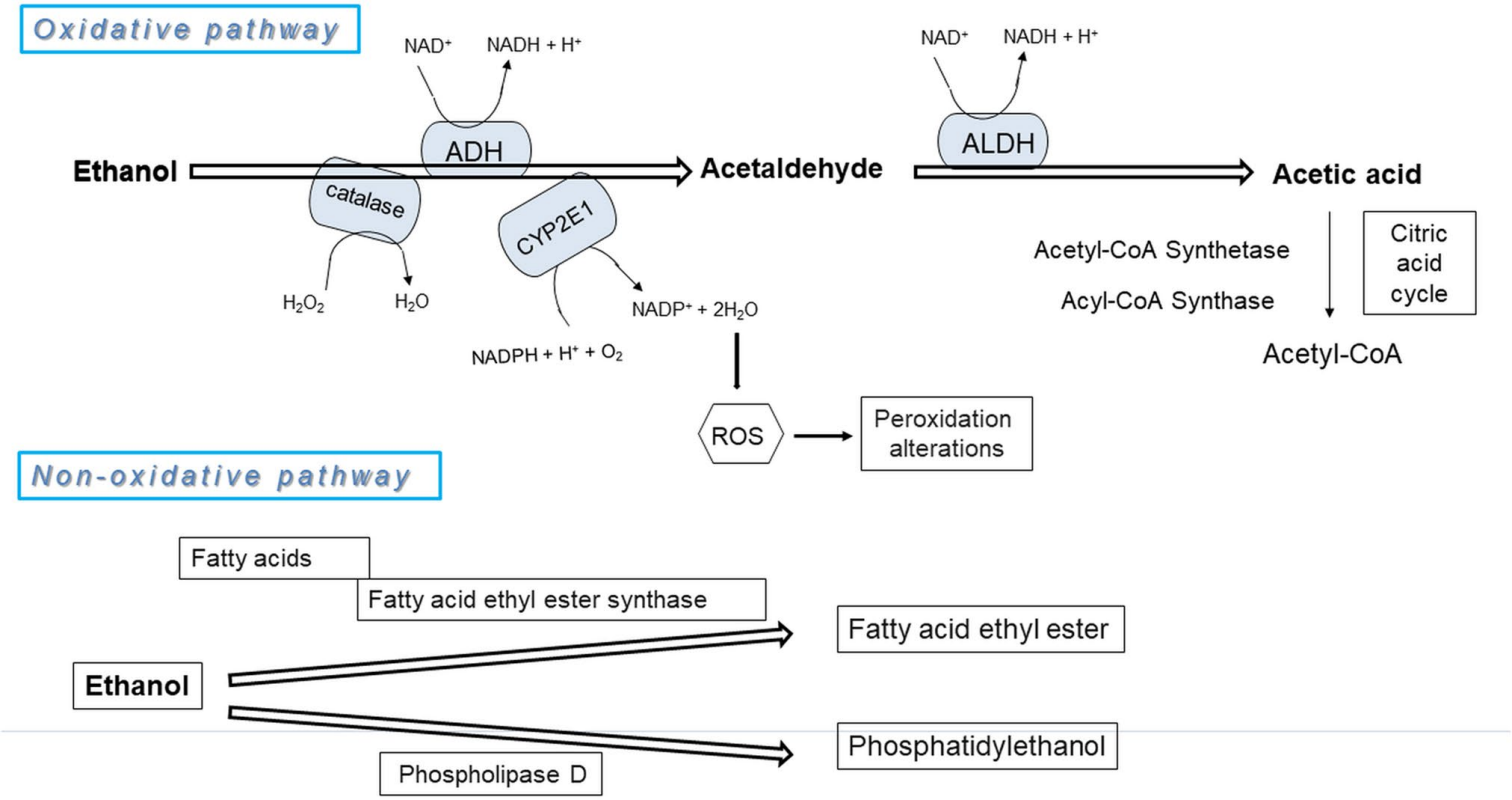
Alcohol oxidative metabolism diagram. *ADH* alcohol dehydrogenase, *ALDH* aldehyde dehydrogenase, *CYP2E1* cytochrome P4502E1, *ROS* reactive oxygen species

The biotransformation of ethanol leads to the generation of metabolites that may cause cell function disturbance or cell death. Ethanol, as an enzymatic substrate, activates oxidative metabolic pathways or is incorporated into different chemical structures in non-oxidative reactions (Fig. 1). The oxidative metabolism of ethanol is predominant [11].

Ethanol metabolism proceeds via a two-stage process. In the first stage, acetaldehyde is created as a dominant metabolite; during the second stage, acetic acid is generated. The first stage of alcohol metabolism to acetaldehyde is catalysed by alcohol dehydrogenase (ADH, EC 1.1.1.1), cytochrome P4502E1 (CYP2E1, EC 1.14.14.1), and catalase (CAT, EC 1.11.1.21). In addition, ethanol is biotransformed to acetaldehyde and free radicals by molybdoflavoproteins, i.e. , xanthine dehydrogenase (XDH, EC 1.17.1.4), xanthine oxidase (XO, EC 1.17.3.2), and/or aldehyde oxidase (AO, EC 1.2.3.1). Incubation of the cytosolic fraction of rat breast tissue containing XDH and XO with cosubstrates (e.g. , NAD^+^, hypoxanthine, xanthine, and its derivatives) significantly enhanced the metabolism of ethanol to acetaldehyde. This process is inhibited by allopurinol, but not by pyrazole [12].

ADH is a cytosolic enzyme that shows broad substrate specificity and is present in many tissues. ADH requires zinc to stabilize the enzyme-active centre. The oxidation of ethanol utilizes the coenzyme NAD^+^ and therefore increases the NADH/NAD^+^ proportion [13]. This enzyme is inhibited by pyrazole and methylpyrazole.

CYP2E1 converts ethanol to acetaldehyde and then to acetic acid. CYP2E1 is localized in liver mitochondria instead of the endoplasmic reticulum. It has been shown that CYP2E1 from mitochondria is more effective in the production of reactive oxygen species (ROS) and cellular damage. The role of CYP2E1 in alcoholic cancerogenesis is complex in that this enzyme is induced by ethanol, but ethanol is also an inhibitor of this enzyme [14].

The induction of CYP2E1 by ethanol leads to the inactivation of retinoic acid (RA). Incubation of RA with the liver microsomal fraction from ethanol-treated rats resulted in the disappearance of RA and an increase in its polar metabolites, 18-hydroxy-RA and 4-oxo-RA levels. Both CYP2E1 antibody and chlormethiazole and allyl sulphide, specific inhibitors of CYP2E1, exert inhibitory effects on RA metabolism in the liver of rats. The metabolism of RA into polar metabolites was completely abolished by disulphiram and liarozole, nonspecific CYP2E1 inhibitors [15].

Individuals differ in their ability to metabolize ethanol through genetic differences in ADH, the enzyme that catalyses the oxidation of approximately 80% ethanol to acetaldehyde in the liver. People differ in their ADH genotype [16]. Genetic features may play an important role in alcoholic breast tumourigenesis.

There are several categories of human ADH isoenzymes. The category I ADH enzymes, the products of ADH1A, ADH1B, and ADH1C genes (in the past named ADH1, ADH2, and ADH3, respectively), mostly participate in the biotransformation of ethanol. Among the category I ADH genes, especially ADH1B and ADH1C, polymorphic variants exist [17]. Among Asians, ADH1B polymorphisms have been found. ADH1C polymorphisms are mainly seen among Caucasians [18]. There are two polymorphisms in ADH1C, i.e. , Ile^350^Val and Arg^272^Gln, which determine the ADH1C*1 allele. ^350^Ile and ^272^Arg define the ADH1C*1 allele, whereas ^272^Gln and ^350^Val determine the ADH1C*2 allele [19]. Persons with ADH1C1-1, ADH1C1-2, and ADH1C2-2 are defined as fast, intermediate, and slow metabolizers of ethanol, respectively [20].

The ADH1C gene is critical in cancer development because it has two alleles, a highly active allele ADH1C*1 and the less active allele ADH1C*2. It was demonstrated that the ADH1C*1 allele plays a crucial role in breast cancer risk in premenopausal women [21]. In a study of 315 women with diagnosed breast cancer and 356 control subjects, the risk of breast cancer was higher in persons carrying two copies of the ADH1C*1 allele, i.e. homozygous for ADH1C*1 than in those carrying only one or no copies of this allele, i.e. , heterozygous or homozygous for the ADH1C*2 allele. In addition, premenopausal women who were homozygous for ADH1C*1 and consumed higher amounts of alcohol had a greater risk of breast cancer than women whose alcohol consumption was moderate (odds ratio, OR = 3.6; 95% CI 1.5–8.8) [22].

In a study of 1047 breast cancer individuals and 1101 control subjects, it was demonstrated that breast cancer risk for fast metabolizers was strongly marked among premenopausal women. The OR in these women was 2.9 (95% CI 1.2–7.1), whereas in postmenopausal women, the OR was 1.8 (95% CI 0.9–3.8) [16]. These data support the opinion that fast metabolizers of alcohol are at higher breast cancer risk from alcohol consumption than slow metabolizers.

Epidemiological studies revealed that ADH1C alleles that lead to the accumulation of acetaldehyde in organisms can increase the risk of alcohol-derived cancers in the mammary gland. In a meta-analysis of a total of 6,159 cases and 5,732 controls in Caucasians, the pooled OR with 95% CI for breast cancer risk connected with ADH1C genotype was evaluated. The lack of an increased breast cancer risk was found in all genetic models (ADH1C1-2 vs. ADH1C2-2: OR = 1.07, 95% CI 0.97–1.19; ADH1C1-1 vs. ADH1C2-2: OR = 1.16, 95% CI 0.94–1.43; dominant model: OR = 1.07, 95% CI 0.97–1.18; recessive model: OR = 1.06, 95% CI 0.93–1.20). In the additive model, where patients were carrying the ADH1C*1 allele, an increased risk of breast cancer was not observed (OR = 1.01, 95% CI 0.97–1.06). These results indicate that ADH1C polymorphism may not be connected with breast cancer risk in Caucasians [23].

Acetaldehyde is rapidly metabolized into a nontoxic acetate by aldehyde dehydrogenase (ALDH, EC 1.2.1.3) in the liver. This reaction also increases the NADH/NAD^+^ proportion. The liver drives acetate to peripheral tissues, where it is metabolized into acetyl-CoA and then may be included in the tricarboxylic acid cycle or fatty acid synthesis.

ALDH is a mitochondrial and cytosolic heterogeneous enzyme (occurring as an isozyme) that participates in the biotransformation of endogenous and exogenous aldehydes. The mitochondrial ALDHs from the livers of rats, hamsters, and humans exhibit similar properties in the oxidation processes. However, the cytosolic ALDH levels in humans differ significantly from those in rodents. The Km value for acetaldehyde as a substrate for human LDH1 is approximately 12 times greater than those for rodents. In addition, ALDH1 is ten times less sensitive to disulphiram inhibition than rodent cytosolic ALDHs. ALDH2 is the most effective for the oxidation of acetaldehyde. This isozyme has the lowest Km (~ 0.2 µM) towards acetaldehyde as its substrate. It was suggested that ALDH2 is the only enzyme that oxidizes acetaldehyde in humans [24].

ALDH2 is located in the mitochondrial matrix of hepatocytes. ALDH2 participates in detoxifying reactions, including the elimination of endogenous compounds such as 4-hydroxy-2-nonenal and malondialdehyde, the products of lipid peroxidation reactions. ALDH2 is also involved in the elimination of neurotransmitter metabolites such as 3,4-dihydroxyphenylacetaldehyde (DOPAL) and 3,4-dihydroxyphenylglycoaldehyde (DOPGAL) in the central nervous system. There is an inactivating point mutation in ALDH2 (named ALDH2*2) with low enzymatic activity [25]. This enzyme form is responsible for acetaldehyde accumulation in tissues.

In a study of 623 female breast cancer patients and 1845 control subjects from East Asia, it was found that alcohol consumption increased the risk of breast cancer regardless of ADH1B and ALDH2 genotypes. The polymorphism of ALDH2 was independently related to elevated breast cancer risk (OR = 1.27; 95% CI 1.02–1.58). The ADH1B polymorphism and the combination of ALDH2 and ADH1B polymorphisms did not show any relationship with breast cancer risk [26].

From the point of view of potential health effects, the existence of the ALDH2*2 mutation in ALDH2, which occurs in approximately 560 million people in East Asia, or almost 8% of the world’s population, is important [27].

Folate and alcohol are dietary factors that exert an impact on the risk of cancer development in humans. Alcoholism is associated with folate deficiency due to limited dietary folate consumption. Heavy alcohol intake decreases folate absorption, increases urinary folate excretion and inhibits key enzymes for one-carbon metabolism. Aberrant DNA methylation, caused by the deficiency of methyl donors, is considered a common negative effect of folate deficiency caused by ethanol. The negative effects of low consumption of nutrients that provide methyl groups and high intake of alcohol are additive. Low methionine and low folate diets coupled with alcohol intake could increase colorectal cancer risk in men [28].

It was suggested that CYP2E1 and ALDH1 enzymes may be critical as causal factors in some tumours, especially in oral cancer. Chronic and high alcohol consumption leads to the induction of CYP2E1, which oxidizes ethanol to acetaldehyde. Acetaldehyde is a well-known carcinogen. This compound interferes with DNA methylation, synthesis and repair, and it also binds to protein and DNA to form stable adducts. It was found that S-adenosylmethionine (SAM), which is a product of folate biotransformation, regulates the catalytic activity of CYP2E1. It was hypothesized that high concentrations of folate may result in an increase in SAM levels, which inhibits the activity of CYP2E1 and causes a diminished production of acetaldehyde from ethanol. Within the ALDH1 family, two enzymes are critical in both ethanol and folate metabolism. ALDH1A1 transforms acetaldehyde into its nontoxic product, acetate. ALDH1L1, known as folate dehydrogenase (FDH), participates in the production of nucleotides. ALDH1L1 is downregulated in some physiological and pathological situations. On the other hand, its upregulation can cause antiproliferative effects. The other hypothesis states that folate interacts with one of the three known response elements that regulate gene expression to upregulate ALDH1A1 and ALDH1L1 expression to reduce acetaldehyde levels and ensure DNA stability [29].

The electrophilic character of acetaldehyde ensures the possibility of participation in reactions with some endogenous compounds to produce various products, such as salsolinol. Salsolinol (6,7-dihydroxy-1-methyl-1,2,3,4-tetrahydroisoquinoline) is a product of the condensation reaction between acetaldehyde and dopamine. This compound, widely occurring in many edibles, did not show any necrotic effect on SH-SY5Y cells in vitro*.* This product exerted a neuroprotective effect against the neurotoxin 6-hydroxydopamine. Salsalinol significantly reduced the reactive oxygen species level in SH-SY5Y cells treated with H_2_O_2_ as well as the caspase activity. Serum levels of tumour necrosis factor-alpha (TNFα) and C-reactive protein (CRP) both served as indirect markers of neurotoxicity in salsolinol-treated rats and were not significantly different from the control animals [30].

Acetaldehyde reacts with amino groups and forms Schiff bases, altering the structures and functions of many proteins, including alkaline histones [31]. Acetaldehyde inhibits retinoic acid (RA) biosynthesis. RA is produced from retinaldehyde by retinaldehyde dehydrogenase (RALDH2, ALDH1A2). RALDH2 shows more affinity for acetaldehyde as a substrate than for retinaldehyde [32].

Acetaldehyde is carcinogenic in rodents and causes sister chromatid exchanges as well as chromosomal aberrations in human cells. The well-known adduct from acetaldehyde is N^2^-ethyl-2'-deoxyguanosine, which is present in liver DNA obtained from ethanol-exposed rodents and in leucocytes obtained from human alcohol consumers. The carcinogenic relevance of this adduct is unclear because of the lack of evidence supporting its mutagenic activity in mammalian cells. Another DNA adduct, 1,N^2^-propano-2'-deoxyguanosine (PdG), can also be generated from acetaldehyde in the presence of histones and other basic compounds. PdG is responsible for genotoxic and mutagenic effects [34]. In vitro studies show that acetaldehyde induces clastogenic effects, as evidenced by the increase in micronuclei frequency, DNA breaks, the cell growth cycle stopping at the G2/M phase, and the decrease of cell vitality [33, 34].

Acetaldehyde reactivity also leads to covalent alterations in lipids and proteins by forming different adducts. Acetaldehyde lessens glutathione (GSH) levels and, consequently, cellular redox equilibrium by different mechanisms. These mechanisms may include, among other things, the nonenzymatic binding of GSH, a decrease in glutathione peroxidase activity, destruction of GSH into cysteinyl glycine, and disturbances of the transsulphuration process [35].

GSH serves to protect cells against endogenous and exogenous electrophiles. This tripeptide is a cofactor for enzymes that metabolize H_2_O_2_ and lipid peroxides. GSH is connected with different electrophilic compounds by glutathione S-transferase (GST). The products of these reactions are well eliminated from organisms. GSH biosynthesis is linked to the cellular methylation process through the transsulphuration pathway. The methylation process is essential for epigenetic gene regulation [35].

Apart from oxidation, there are several non-oxidative reactions of ethanol that lead to the enzymatic conjugation of this chemical to endogenous metabolites such as glucuronic acid, sulphate phospholipids, and fatty acids. Ethyl glucuronide, ethyl sulphate, phosphatidylethanol and fatty acid ethyl esters are the products of these reactions. These conjugation reactions quantitatively represent a minor part of the biotransformation processes of ethanol in comparison with the oxidative reactions. These last reactions may have pathological significance due to the modification of metabolic processes [36, 37].

Ethanol causes metabolic alterations that may lead to cancer development. According to the International Agency Research on Cancer (IARC), alcoholic beverages and acetaldehyde are classified in Group 1, i.e. substances carcinogenic to humans [2].

## Alcohol effects on breast cancer

Studies of alcohol in relation to breast cancer incidence have included hundreds of thousands of women. Evidence is consistent that alcohol consumption, even the intake of less than 10–50 g per day, leads to an increased risk of this disease. In addition, evidence shows that possible early indicators of risk, such as benign breast disease and increased breast density, are associated with alcohol consumption. Evidence is less strong for differences based on geographic region, beverage type, drinking pattern, or breast cancer subtype. Knowledge of breast cancer caused by alcohol as a risk factor is low in society [5].

There are numerous risk factors for breast cancer. In a case–control study conducted in Poland, significant associations between some risk factors and breast cancer were observed. Obesity caused an elevated risk of mammary gland cancer in women compared to subjects with a BMI < 30 (OR = 1.9; 95% CI 1.16–3.04). Alcohol consumption ≤ 15 years of life led to a duplication of breast cancer risk (OR = 1.98; 95% CI 1.06–3.69). Women who breastfed for less than 3 months had a higher breast cancer risk (OR = 2.3; 95% CI 1.52–3.5). Women with basic education (OR = 2.5; 95% CI 1.49–4.19) and those living in rural areas (OR = 1.7; 95% CI 1.05–2.9) had an increased breast tumour risk [38].

In women, low to moderate alcohol intake increases the risk of some tumours. Every extra drink (a unit) systematically consumed per day is responsible for 11 breast cancers per 1000 women up to the age of 75 years. Increased alcohol drinking leads to an elevated risk of mammalian tumours (12%; 95% CI 9–14%) (*p* trend < 0.001). The types of alcoholic beverages do not influence the cancerogenic risk level [39].

In a case–control study of 243 cases and 423 control subjects, it was found that women with a prior ductal carcinoma in situ diagnosis were at higher risk of invasive breast cancer. It was observed that drinkers with ductal carcinoma in situ*,* consuming at least one alcoholic drink per day, had a higher risk of invasive breast cancer (OR = 1.79; 95% CI 1.01–3.17). The risk of this cancer type in women was not significantly associated with smoking [40].

The relationship between alcohol consumption and postmenopausal breast cancer risk among 87,724 women in the Women’s Health Initiative Observational Study prospective cohort from 1993 through 1998 was evaluated. A total of 2944 women with invasive breast cancer were diagnosed during follow-up through September 15, 2005. Alcohol consumption was positively related to the risk of invasive breast cancer overall, invasive lobular carcinoma, and hormone receptor-positive tumours. Women who consumed seven or more alcoholic beverages per week had an almost twofold increased risk of hormone receptor-positive invasive lobular carcinoma (hazard ratio, HR = 1.82; 95% CI 1.18–2.81), but not a statistically significant increased risk of hormone receptor-positive invasive ductal carcinoma (HR = 1.14; 95% CI 0.87–1.50) compared with non-drinkers. The difference in HRs per drink per day among current drinkers was 1.15 (95% CI 1.01–1.32). The absolute rates of hormone receptor-positive lobular cancer among non-drinkers and current drinkers were 5.2 and 8.5/10,000 person-years, respectively, whereas for hormone receptor-positive ductal cancer, they were 15.2 and 17.9/10,000 person-years, respectively [41].

Different patterns of alcohol drinking may have different effects on breast cancer development even when the total amount of alcohol intake is constant. The study was performed on a group of 9577 women (mean age 34 years), with a median follow-up of 11.8 years. Among 104,932 women-years of follow-up, 88 cases of breast cancer were diagnosed. Women in the binge drinking group had a higher risk of breast cancer (HR = 1.76; 95% CI 1.03–2.99) than those in the non-binge drinking category. In the stratified analysis, a twofold higher risk for premenopausal breast cancer was associated with a binge drinking habit (HR = 2.06; 95% CI 1.11–3.82) [42].

In the European Prospective Investigation into Cancer and Nutrition (EPIC) study, including a cohort of more than 360,000 women from 10 countries in Europe, the association between alcohol consumption and the risk of breast cancer was stronger for women with oestrogen receptor-positive tumours than for those with oestrogen receptor-negative tumours [43].

## Alcohol effects on oestrogen levels and oestrogen receptors

The relationship between alcohol consumption and oestrogen and androgen concentrations in the blood of postmenopausal women was evaluated in a cross-sectional study. It was found that women who consumed over 25 g of alcohol per day had higher levels of oestrone (E_1_) and oestradiol (E_2_). The concentrations of dehydroepiandrosterone sulphate (DHEAS), testosterone, and sex hormone-binding globulin (SHBG) were not changed compared to those in non-drinking women [44]. In another study, when postmenopausal women consumed 15 or 30 g alcohol per day for 8 weeks, they had an elevated concentration of oestrone sulphate in serum, increasing by 7.5% (95% CI  − 0.3 to − 15.9%) and 10.7% (95% CI 2.7–19.3%), respectively. DHEAS concentrations also increased by 5.1% (95% CI 1.4–9.0%) and 7.5% (95% CI 3.7–11.5%), respectively, relative to levels in women who consumed the placebo [45].

In a similar study at week 4, when the women consumed 15 g of alcohol per day, oestrone sulphate in serum increased by an average of 6.9% (not significantly). When these women drank 30 g of alcohol per day, oestrogen was elevated by an average of 22.2% (significantly). Additionally, DHEAS levels were increased (significantly) by an average of 8.0% and 9.2% after intake of alcohol at doses of 15 g and 30 g daily, respectively. The concentrations of serum oestrone sulphate at week 4 versus week 8 across both doses of alcohol were similar. However, DHEAS concentrations were elevated significantly from week 4 to week 8 for all alcohol doses administered. Thus, the hormonal effects due to moderate alcohol intake are seen early, within 4 weeks from the start of alcohol consumption [46].

A controlled-diet study lasting for six consecutive menstrual cycles in women with a history of regular menstrual cycles who consumed 30 g of ethanol (i.e. approximately two average drinks) per day was conducted. Plasma DHEAS levels were 7.0% higher in the follicular phase (*p* = 0.05) than in the no alcohol group. In the peri-ovulary phase, increases of 21.2% (*p* = 0.01) in plasma E1 levels, 27.5% (*p* = 0.01) in plasma E2 levels, and 31.9% (*p* = 0.009) in urinary E2 concentrations were observed. In the luteal phase, E1 levels in the urine increased by 15.2% (*p* = 0.05), E2 levels rose by 21.6% (*p* = 0.02), and oestriol levels increased by 29.1% (*p* = 0.03). The percent of bioavailable E2, defined by the sum of free E2 and albumin-bound E2, expressed as a percentage, did not change [47].

In MCF-7 human breast cancer cells in vitro, ethanol stimulates cell proliferation and enhances ERα and aromatase expression. This finding supports the role of ER signalling in the proliferation of breast cancer cells. Ethanol has been found to stimulate the proliferation of human ER + but not ER− breast cancer cells in vitro [48]. Moreover, in hormone-responsive MCF-7 and T47D cells, ethanol increased the activity of the liganded ER-α in a dose-dependent manner and caused downregulation of BRCA1 expression, the tumour-suppressor gene [49].

The results of the EPIC study and the Endogenous Hormones and Breast Cancer Collaborative Group have confirmed the relationship between elevated serum concentrations of oestrogens and androgens and low serum levels of SHBP and high premenopausal breast cancer risk. It has been suggested that alcohol drinking increases sex hormone levels in both pre- and postmenopausal females. In the EPIC study, higher levels of both total and free testosterone and lower levels of SHBP were observed in postmenopausal women who consumed over 25 g/day of alcohol [50, 51].

In another study, alcohol consumption was significantly positively associated with plasma luteal concentrations but not with androgen levels or oestrone or oestradiol in the follicular phase of the menstrual cycle [52].

It has been found that higher premenopausal circulating testosterone levels are associated with an increased risk of the development of breast cancer, but a significant association of oestradiol or progesterone with breast cancer risk, overall, by menstrual cycle phase or by tumour receptor status has not been shown; a possible risk increase with higher oestradiol levels for tumours diagnosed before age 50 was seen [53].

The relationship between alcohol consumption and ER + and PR + breast cancer has been shown in numerous epidemiologic studies [8, 9, 43]. In a group of 989 women with histologically confirmed breast cancer and a control group of 1350 women, alcohol drinking was related to ER + tumours (OR = 2.16; 95% CI 1.68–2.76) for the group consuming > or = 13.8 g alcohol per day. For a 10-g increase in alcohol consumption per day, the OR was 1.13 (95% CI 1.07–1.20). For ER− cancers, the association with alcohol intake was not significant (OR = 1.36; 95% CI 0.93–2.01). The effects for ER + PR + tumours were similar to those for all ER + tumours (OR = 2.34; 95% CI 1.81–3.04) for subjects with an alcohol intake of > or = 13.8 g per day. For ER-PR- breast cancer, no significant relationship was observed (OR = 1.25; 95% CI 0.81–1.94) [54].

Alcohol consumption at the highest intake level (> or = 10 g of alcohol per day) was associated with an increased risk of the development of ER + tumours, irrespective of PR status compared to non-drinkers (RR = 1.35; 95% CI 1.02–1.80 for ER + PR + tumours and RR = 2.36; 95% CI 1.56–3.56 for ER + PR −  tumours). The absolute rate of ER + breast cancer was 232 per 100,000 person-years among women in the highest category of alcohol consumption and 158 per 100,000 person-years among non-drinkers. No association between alcohol consumption and the risk of developing ER − tumours was observed. In addition, it was observed that there is a statistically significant interaction between alcohol consumption and the use of postmenopausal hormones and the risk for ER + PR + tumours [55].

Oral ethanol administered at a dose of 0.225 g/kg body weight increased serum oestradiol levels significantly by 27–38% in premenopausal women, especially in the midphase of the menstrual cycle [56]. It is well known that oestrogens may cause breast cancer by direct actions on the ER and as a consequence of their oxidation leading to reactive metabolites [57].

In general, it has been confirmed that the consumption of alcohol is more strongly related to ER + breast cancers than ER− breast cancers [54, 55].

Finally, it has been reported that in vitro*,* culturing ER + human breast cancer cells in ethanol-containing medium was associated with an increase in their proliferation rate, ERα content and ER transcriptional activity. Since these changes were not observed in ER- breast cancer cells and because alcohol intake has been associated with an increased level of circulating oestrogens, it has been postulated that aromatase expression could be increased following ethanol exposure. The study shows a 1.3-fold increase in cell proliferation after 6 days of culture of MCF-7 cells in the presence of 0.1% ethanol. After a 6-day exposure to 0.1% ethanol, there was a 1.7-fold increase in ERα mRNA (not significant) and a 3.3-fold increase in ERα content (significant). In addition, a 2.4-fold increase in the aromatase mRNA level was demonstrated. These results are in agreement with the involvement of ER signalling in ethanol-induced stimulation of breast cancer cell proliferation [48]. Moreover, exposure of MCF-7 breast cancer cells to ethanol induced an increase in the mRNA levels of two well-known oestrogen target genes, PR and pS2. This result was confirmed by an increase in luciferase activity in pEREtkLuc-transfected MCF-7 cells exposed to ethanol. These effects, whose intensity was similar to those of oestradiol, were also observed in a steroid-free medium and were inhibited by the anti-oestrogen (ICI 182,780). This suggested a ligand-independent activation of ERα in ethanol-treated cells [58].

## Molecular mechanisms of alcohol-mediated carcinogenesis

**Fig. 2:**
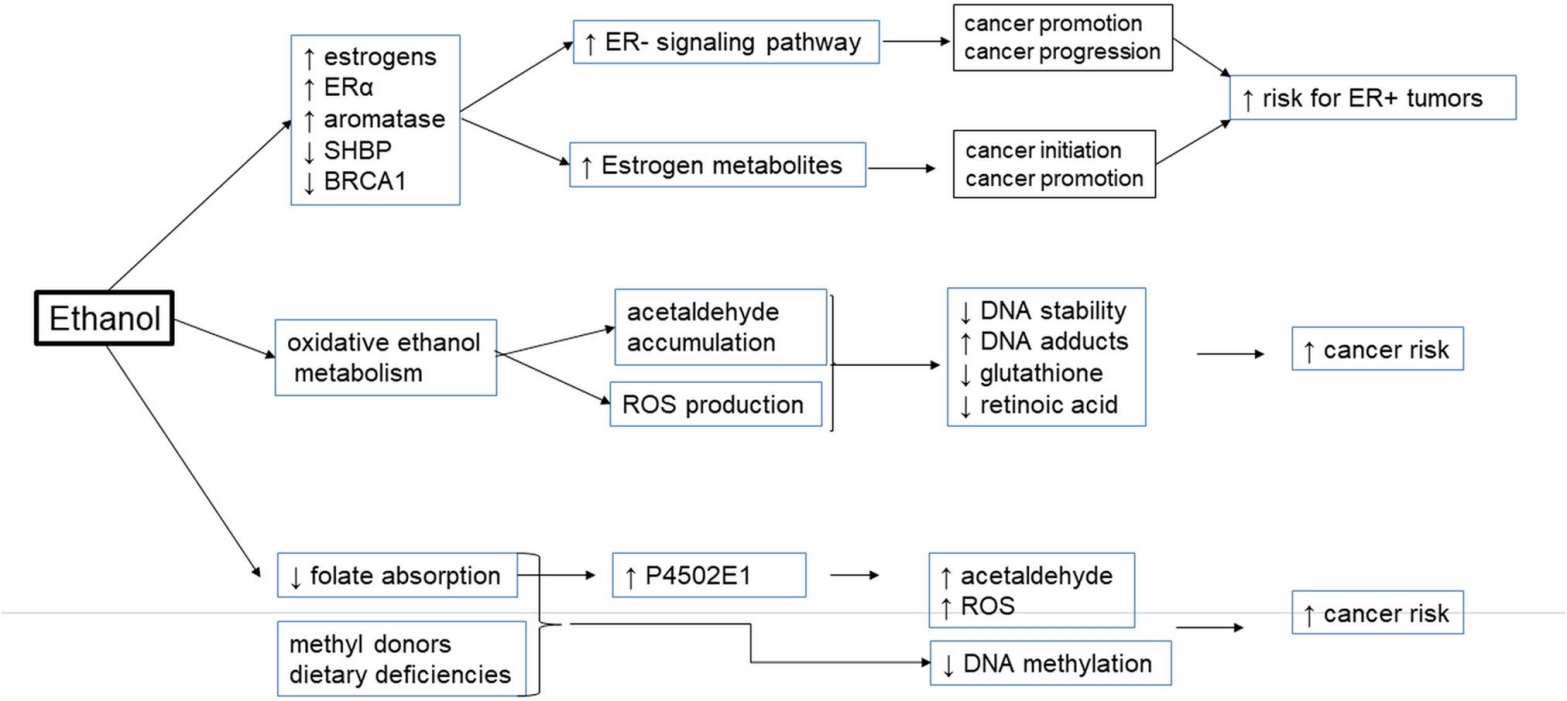
The main mechanisms of alcohol carcinogenicity. *ERα* − oestrogen receptors; *SHBP* sex hormone-binding globulin, *BRCA1* tumour-suppressor gene, *ROS* reactive oxygen species, *ER* + *tumours* tumours having oestrogen receptors, *P4502E1* cytochrome P4502E1

Despite consistent evidence linking breast cancer to alcohol consumption, the mechanisms for carcinogenesis induced by alcohol are not completely known. Several ethanol actions have been hypothesized (Fig. 2). These include alcohol effects on oestrogen concentrations and their receptors, the production of acetaldehyde or reactive oxygen species (ROS), and reduction in the absorption of essential nutrients. Ethanol may also cause carcinogenic effects by disturbing DNA methylation and retinoid metabolism [3].

Alcohol-associated carcinogenesis may be caused by smoking, diet, comorbid diseases, and genetic predisposition. An increase in endogenous oestrogen levels by alcohol drinking may lead to breast cancer. Patients with, among other things, some chronic liver diseases, gastroesophageal reflux disease and colorectal polyps are particularly susceptible to the tumourogenic action of ethanol. Carriers of the ALDH2*2 allele have a higher probability of alcohol-related oesophageal cancer. Carriers of ADH1C*1 homozygotes and methylenetetrahydrofolate reductase (MTHFR) 677CT variants are also at higher risk for alcohol-connected tumours. Factors such as poor oral hygiene, deficit of folate, vitamin B6, or methyl donors in diet or an excess of vitamin A and β-carotene in a meal also increase the risk of alcohol-associated tumours [59].

In vitro studies using human tumour cells have identified signalling molecules that may contribute to the effects of alcohol, including ROS, acetaldehyde, matrix metalloproteases, ErbB2/Her2/Neu receptor tyrosine kinase, cytosolic protein kinases, adenylyl cyclase, E-cadherins, oestrogen receptors, and different transcription factors. The existing data suggest that the epidermal growth factor receptor (EGFR) tyrosine kinase may contribute to breast cancer development and progression [60]. Amplification of ErbB2 receptor tyrosine kinase is observed in 20–30% of women with breast cancer. ErbB proteins that function as receptor kinases include EGFR/ErbB1, ErbB2/neu, ErbB3, and ErbB4. In particular, ErbB2 plays an essential role in the activities of ErbB. Ethanol stimulates invasion by breast cancer overexpressing ErbB2. It was found that overexpression of ErbB2 is positively related to elevated levels of matrix metalloproteinase-2 (MMP-2) and MMP-9. The activation of these metalloproteinases is dependent on c-Jun N-terminal kinases (JNK) and ROS. Ethanol-stimulated cell invasion is significantly inhibited by selective inhibitors of MMP-2. MMP-2 is predominantly expressed in stromal fibroblasts. MMP-2 is also activated by ethanol. The medium collected from ethanol-treated fibroblasts markedly stimulated the invasion of breast cancer cells. In MCF-7 human neoplastic cells treated with ethanol in vitro*,* higher amounts of active MMP-2 and MMP-9 in their culture medium were observed. E2 induces growth in MMP-2 and MMP-9 levels. The anti-oestrogen ICI 182,780 inhibits the E2-induced increase in MMP-2 and MMP-9 secretion. In the case of ethanol exposure, this ER antagonist was only powerful on MMP-9 secretion. Although MMP-9 transcription in MCF-7 breast cancer cells was not sensitive to E2 or ethanol, MMP-2 transcription was stimulated by these compounds [61–63].

It has recently been shown that epithelial-mesenchymal transition (EMT), a process by which cancer cells invade and migrate, and loss of cell‒cell adhesion molecules such as E-cadherin which have an increased level of mesenchymal proteins, e.g. vimentin, play a significant role in cancer progression and metastasis. Alcohol upregulated an EMT marker, vimentin, in colon and breast cancer cells. In colon and MCF-7 and MDA-MB-231 breast cancer cells, this compound caused an increase in MMP-2, MMP-7, and MMP-9 levels as well as cell migration. Alcohol also stimulated nuclear localization of a transcription factor (Snail) involved in tumour progression and malignancy. The snail level was significantly elevated in colonic tissue from alcoholics. Moreover, it was found that alcohol increased EGFR activation. EGFR inhibitors (e.g. AG1478 and TAPI-2) blocked Snail mRNA expression and alcohol-induced cell migration. These data indicate that cancer progression induced by alcohol through EMT activity is associated with an EGFR–Snail-mediated pathway [64].

Alcohol stimulates mammary tumour growth by activating vascular endothelial growth factor (VEGF). In a mouse xerograph model of mammary tumours and a three-dimensional (3D) tumour/endothelial cell coculture system, it was discovered that alcohol stimulated tumour angiogenesis and accelerated tumour growth. In addition, alcohol induced VEGF expression in breast cancer cells in vitro and in vivo. The inhibition of VEGF by a specific inhibitor reduced both angiogenesis of tumours and alcohol-promoted tumour growth [65]. VEGF not only induces angiogenesis but also uncouples endothelial cell‒cell junctions and causes vascular permeability and oedema, resulting in extensive injury to ischaemic tissues after stroke or myocardial infarction. In cancer, VEGF-mediated disruption of the vascular barrier may cause tumour cell extravasation, leading to metastasis [66].

Metastasis is a process that includes intravasation and extravasation of cancer cells. The integrity of the vascular endothelial barrier plays a pivotal role in these processes. Ethanol at a concentration of 200 mg/dl disrupted endothelial monolayer integrity. This effect was reversible after the withdrawal of ethanol. The disruption of endothelial monolayer integrity led to growth in the invasion of cancer cells. Ethanol induces the endocytosis of VE-cadherin. VE-cadherin is an important component of the adherens junction and is responsible for vascular endothelial integrity. These results suggest that ethanol may simplify cancer metastasis by disjunction of the vascular endothelial monolayer [67].

In breast cancer cells (MCF-7, BT20, and T27D) chronically exposed to ethanol in vitro*,* increased aggressiveness of these cells by the mitogen-activated protein kinase (p38γMAPK/RhoC) pathway was observed. In this type of exposure to ethanol, a scattering of the examined cell colonies was observed. Moreover, increased colony formation and stimulation of cell invasion/migration were found. In addition, the population of cancer stem-cell like cells (CSCs) increased. The above-mentioned cancer cells treated with ethanol in vitro manifested a higher increase in velocity and metastasis in mice in vivo [68].

p38γ is one of the isoforms of a p38 MAPK enzyme that includes the following isoforms: p38α, p38β, p38γ and p38δ. These p38 MAPKs are kinases of serine/threonine. These enzymes are activated by different cellular and environmental factors, as well as other inflammatory cytokines. It was found that p38γ MAPK may participate in carcinogenesis. However, the molecular mechanism of its action is unknown. It was shown that p38γ MAPK leads to EMT growth in breast cancer cells. EMT facilitates cancer cell progression and metastasis and participates in the regulation of CSCs. CSCs have a self-renewal capacity and are resistant to chemotherapy. While overexpression of p38γ MAPK activated EMT, its downregulation depressed EMT. Whereas p38γ MAPK increased the CSC number, knockdown of p38γ MAPK decreased the CSC ratio in breast cancer cells. MicroRNA-200b (miR-200b) was downstream of p38γ MAPK and negatively regulated by p38γ MAPK. miR-200b inhibited p38γ MAPK-induced EMT, while miR-200b inhibitors stimulated EMT. The regulation of miR-200b expression by p38γ MAPK proceeds through inhibiting GATA3. GATA3 is a transcription factor that regulates miR-200b expression. p38γ MAPK induces GATA3 and leads to its proteasome-dependent degradation; p38γ MAPK participates in the regulation of the CSC population, migration/invasion, tumourigenesis and cell transformation. EMT plays a crucial role in cancer progression, the dissemination of cancer cells from solid tumours and the formation of metastases. During the process of EMT, epithelial cancer cells achieve molecular characteristics that facilitate the loss of epithelial features and acquisition of the mesenchymal phenotype. Such a transformation leads to cancer cell migration and invasion [69–71].

Alcohol causes migration and invasion of triple-negative breast cancer cells (TNBC) through activation of p38 MAPK and JNK. These cells are a subtype of breast tumours that lack ER and PR hormone receptors and HER2 receptor expression. It has been shown that alcohol at low concentrations (0.025–0.1%) in MDA-MB-231 and MDA-MB-468 cell lines caused cell proliferation, migration, and invasion. Both of these cell lines represent TNBC. The changes observed were induced by ROS production, activation of p38 MAPK, and JNK phosphorylation. Alcohol exposure activated the nuclear transcription factor NF-кB and increased the transcription of NF-кB-targeted genes. In TNBC cells, acetaldehyde, a major alcohol metabolite, induced cell migration and invasion and increased the phosphorylation of p38, JNK, and NF-кBα in a manner similar to ethanol [72].

## Prevention

The induction and development of female breast cancer are conditioned by different factors. These factors include alcohol consumption, precocious puberty, late menopause, first pregnancy in late age, a short period of breastfeeding, use of combined oestrogen and synthetic progestogen hormonal therapy, a high-fat diet, and little or lack of physical exercise. The elimination of some of these factors, as a primary prevention, may protect against morbidity and mortality caused by breast cancer [73, 74]. The diagnostic methods, e.g. mammography, ultrasonography, automated ultrasound, and palpable breast examination, as a secondary prevention, may contribute to the early detection of cancer or precancerous alterations [75, 76].

Some compounds, particularly phytochemicals present in food, drinks, and dietary supplements, which have potent inhibitory activity on the enzymes of ethanol metabolism to acetaldehyde [77], may be recognized as both primary and secondary prevention agents. Antineoplastic drugs may be considered secondary prevention factors.

## Phytochemicals

Actein, as a natural compound, is obtained from the root of *Cimicifuga* species. Actein treatment of MDA-MB-231 human breast cancer cells (oestrogen receptor negative) in vitro significantly reduced cell proliferation, migration, and motility, caused G1 phase cell cycle arrest and suppressed MMP protein expression. Moreover, actein blocked the adhesion of breast cancer cells to collagen and decreased the level of integrins. Exposure to this compound led to downregulation of the levels of EGFR, AKT and NF-κB signalling proteins. The in vivo study demonstrated that actein significantly reduced the number of zebrafish embryos with migrated cells by 74% and decreased the number of migrated cells in embryos. Actein is a novel anti-migration natural compound with anti-metastatic activity [78].

Several phytochemicals, especially polyphenols, exert inhibitory action on enzymes responsible for acetaldehyde biosynthesis present in mammary tissue [79–81]. The polyphenolic compounds occurring in plants have antioxidative and possibly anticarcinogenic properties. The inhibition of tumourigenesis has been observed among other compounds, such as phenolic acids, tea and catechins, isoflavones, quercetin, curcumin, genistein, and other flavonoids, resveratrol and lignans. Polyphenols may inhibit carcinogenesis at all stages. Isoflavones and lignans may delay tumour formation by affecting oestrogen-associated activities [82, 83]. An especially attractive polyphenol is ellagic acid because it inhibits oestrogen-mediated mammary carcinogenesis in rats [84]. In addition, foliate was observed to be crucial in the prevention of breast cancer [85].

ALDH activity in the mammary gland is several times lower than that in the liver. Mitochondrial ALDH activity is subject to the inhibitory action of 4-hydroxy-2-nonenal, the lipid peroxidation byproduct, which is both a substrate and an inhibitor of this enzyme [86]. Thus, withdrawal of acetaldehyde from breast tissue is more difficult than from the liver. Moreover, the activity of the reduced glutathione/glutathione S-transferase system, which detoxifies acetaldehyde, is low in breast tissue [87].

The administration of cysteine may diminish acetaldehyde accumulation in the mammary gland by generating 2-methylthiazolidene-4-carboxylic acid, a stable adduct [88]. N-Acetylcysteine (NAC) can be given instead of cysteine because of its lower toxicity. NAC is a precursor of GSH. NAC exerts preventive action by antioxidant activity and inhibition of DNA adduct formation [89]. Additionally, resveratrol, a natural phenolic antioxidant with stilbene structure and dihydrolipoic acid, can counteract the generation of depurating oestrogen-DNA adducts [89].

The carcinogenic effect of ethanol may also exist as the result of catechol-O-methyltransferase (COMT) detoxifying pathway dysfunction. COMT participates in the conjugation of catechol oestrogens in the presence of S-adenosylmethionine (SAM), folate, and vitamins B6 and B12. SAM administration may diminish oxidative stress by GSH synthesis, attenuate the inflammatory process by decreasing TNF-α levels, and increase the biosynthesis of interleukin-10 [90].

In the transmethylation reaction, S-adenosylhomocysteine (SAH) is produced with the participation of SAM. SAH is a potent inhibitor of the methylation reaction. Chronic ethanol drinking depleted the hepatic level of SAM, increased the plasma level of homocysteine and hepatic concentration of SAH, and reduced the folate plasma level in animals and humans. Administration of SAM causes the restoration of the hepatic level of GSH reduced by alcohol [90]. SAM administration may be an efficient agent in the prevention of alcoholic mammary cancer.

In the studies of many selected Chinese medical plants, a highly positive correlation between antioxidant capacities, expressed in inhibition of xanthine oxidase (XO) and neutralization of the superoxide radical, and total phenolic content has been found [79, 91, 92]. The hydroxyl groups at C-5 and C-7 and the double bond between C-2 and C-3 of the flavonoid structure were critical for the high inhibitory effect on XO. Flavonoids contain 15 carbonic atoms forming a characteristic structure consisting of two aromatic rings joining the tricarbonic bridge or heterocyclic ring. Flavones were slightly more active at inhibition than flavanols. These compounds have been divided into six groups according to their effect on XO and superoxide radicals: Group A—lack of inhibitory activity on XO and superoxide radical neutralizing capacity; Group B—XO inhibitors without superoxide radical neutralizing capacity; Group C—XO inhibitors with superoxide radical neutralizing capacity; Group D—XO inhibitors with pro-oxidant activity on the generation of superoxide radicals; Group E—compounds with a minimal inhibitory effect on XO, but with a pro-oxidant activity on the generation of superoxide radicals; Group F—flavonoids without effect on both XO and superoxide radicals [79].

In another study, it was found that apigenin, galangin, kaempferol, quercetin, genistein and resveratrol potently inhibited the XO enzyme among 30 bioactive compounds present in the edible food plants tested. Flavonoids exhibit highest, anthocyanins and hydroxycinnamic acids moderate, while maslinic acid, ellagic acid, salicylic acid, [6]-gingerol and flavon-3-ols demonstrated weak XO inhibitory activity. The molecular docking study indicates that these bioactive chemicals bind with the active centre of XO and occupy this centre, which further prevents the entrance of substrate and results in the inhibition of XO [93].

Recently, it was revealed that the inhibitory activity of alk(en)yl phenols, present in many edible plants, towards XO is related to both the hydroxy group arrangement in the phenol moiety and the alk(en)yl chains. The inhibitory activity of these compounds in relation to XO is expressed as a combination of uric acid synthesis inhibition and suppression of superoxide anion (O2^−^) production. The inhibitory activity of alk(en)yl phenols was divided into three types of processes. The first is XO activity inhibition, the second is reduction of O2^−^ production and the third is O2^−^ scavenging [94].

Alkyl gallates, especially octyl-, decyl- and dodecyl-gallates, competitively inhibit the activity of XO expressed by uric acid formation. The inhibition level grows with increasing alkyl chain length. Gallic acid and its esters equally suppress O2^−^ production catalysed by XO. These data indicate that alkyl gallates may lead to the protection of the organism from breast cancer and urolithiasis [95].

There is evidence indicating that a ketogenic diet can delimit glucose availability to tumour cells and thereby extend ketone body levels from fatty acid oxidation. Glycolysis is a main metabolic process for cancer cells that provides these cells ATP molecules necessary for growth in hypoxic conditions. It is suggested that the introduction of a ketogenic diet in the clinic could improve progression in survival among women with breast cancer [96].

## Drugs as prevention factors

Selective oestrogen receptor modulators (SERMs), such as lasofoxifene, raloxifene, and tamoxifen, and aromatase inhibitors, i.e. anastrozole, letrozole, and exemestane, are drugs applied in breast cancer therapy. Tamoxifen and raloxifene reduced the morbidity and mortality from breast cancer. Promising results of aromatase inhibitor tests indicate that these compounds are efficient in the prevention of ER + breast cancer. Instead, present-day investigations are concentrated on elaborating preventive therapies for other subtypes of breast cancer, such as HER2 + and TNBC. HER2 + breast cancers are at present treated with trastuzumab and lapatinib. A few drugs, such as poly(ADP-ribose) polymerase inhibitors, vitamin D, and retinoids, are currently being examined for the prevention of TNBC [97]. The other selected therapies tested include monoclonal antibodies (mAbs), antibody‒drug conjugates (ADCs), tyrosine kinase inhibitors (TKIs), and cyclin-dependent kinase (CDK) inhibitors [98].

Exemestane, an aromatase inhibitor, has been examined for breast cancer prevention in postmenopausal females. In a group of 4,560 high-risk postmenopausal females treated with exemestane 25 mg daily for approximately three years, there was a 65% relative decrease in the annual occurrence of invasive breast cancer when compared with the placebo. In addition, a 53% decrease in invasive plus noninvasive breast cancer was observed. Adverse effects from exemestane are generally mild, with the most common being diarrhoea, joint pain, and menopausal symptoms. It is crucial that exemestane did not increase the risks of endometrial cancer, thromboembolism, cardiovascular disorders, or cataracts. Instead, joint stiffness and arthralgia were observed more frequently than in the case of tamoxifen and raloxifene [99].

It was estimated that practically half of the women at high and moderate risk of breast cancer could benefit from the application of current therapy, i.e. exemestane, raloxifene, antrazole, and tamoxifen. Moreover, controlling body weight, exercise and moderating alcohol consumption in all women could diminish breast tumour risk by approximately 30% [100].

It was found that breast cancer risk increased by 7% to 10% for each additional daily alcohol consumption. A drink is half a pint of 4% alcohol content beer or cider or 25 mL of 40% spirits, while a small 125-mL glass of 12% wine is 1.5 drinks. Females who consume four to nine drinks weekly are 15% more likely to develop breast cancer than non-drinkers [101]. It was suggested that not drinking more than one drink per day in women should minimize breast cancer risk.

Carcinogenic prevention includes not only middle- and late-aged women, but also younger women after menarche [102]. For carcinogenesis in women, the most susceptible period is between menarche and first pregnancy [103].

## Conclusions

This review presents information on important findings concerning ethanol metabolism, ethanol breast cancer development, and its prevention.

Most data indicate that alcohol consumption increases the risk of mammary gland tumour in both pre- and postmenopausal women. Worldwide, the number of deaths from female breast cancer increases. The data indicate that alcohol consumption is mainly involved in the incidence in ER + and/or PR + breast cancer. Increasing alcohol consumption was related to a rise in breast cancer risk. Different manners and amounts of alcohol consumption may have varied influences on breast cancer development.

Ethanol consumption activates metabolic pathways in an oxidative way or incorporates this compound into different chemical structures in non-oxidative reactions. The oxidative metabolism of ethanol predominates over the non-oxidative metabolism. Ethanol metabolism includes a two-stage process. Under the first stage, acetaldehyde, as a primary metabolite, is generated; during the second stage, acetic acid is produced. While the first stage is metabolic activation, the second stage is detoxification.

The metabolism of ethanol is catalysed by numerous enzymes, including ADH, CYP2E1, CAT, XOR, AO, and ALDH. These enzymes most often exist as different classes of isoenzymes that cause differentiation in individual and racial ethanol metabolism and a variety of breast cancer risks.

The mechanisms by which ethanol induces carcinogenesis include oxidative stress, altered DNA methylation and interaction with retinoid metabolism. In addition, signalling molecules such as acetaldehyde, ROS, matrix metalloproteinases, cytosolic protein kinases, E-cadherins, and different transcription factors may contribute to the carcinogenic effects of ethanol. EGFR tyrosine kinase may contribute to breast cancer development and progression. Ethanol stimulates invasion of breast cancer by overexpression of ErbB2, which is positively associated with elevated levels of matrix metalloproteinases. The activation of metalloproteinases is dependent on c-Jun, JNK, and ROS. Ethanol activates VEGF and EMT, which play a significant role in cancer progression and metastasis. This xenobiotic causes migration and invasion of triple-negative breast cancer cells through activation of p38MAPK and JNK. Ethanol activates the nuclear transcription factor and increases the transcription of NF-κB-targeted genes.

There are primary and secondary prevention strategies that may improve rates of morbidity and mortality from breast cancer. Some phytochemicals present in food, drinks, and dietary supplements that exert an inhibitory effect on the enzymes of ethanol metabolism to acetaldehyde may be recognized as prevention agents. The administration of cysteine, NAC, and SAM may diminish acetaldehyde accumulation in mammary tissues and may stimulate catechol oestrogens’ conjugation by COMT. The compounds that exert their action on the ER and aromatase blocking agents inhibit the ER or oestrogen-activated pathways. The selective therapies currently tested include tyrosine kinase inhibitors, cyclin-dependent kinase inhibitors, antibody‒drug conjugates, and monoclonal antibodies.

## Data Availability

Data sharing is not applicable to this article as no datasets were generated or analysed during the current study.
